# A Petrified Bean in the Urethra—Removed by Incision

**Published:** 1851-05

**Authors:** 

**Affiliations:** Meerane


*A Petrified Bean in the Urethra—Removed by Incision.* By Dr. Leopold, of Meerane.—On the 16th December, 1848, Dr. Leopold was summoned to a child, four years of age, suffering under the symptoms of stone in the bladder. The father obstinately refused to permit a catheter to be passed; internal remedies were therefore administered to endeavour to allay his sufferings, but in vain. Examination by the rectum failed to give indication of stone in the bladder. A hard substance, the size of a bean, could be felt in the urethra behind the scrotum, and could be moved only very slightly backwards and forwards. The catheter, when at last allowed to be passed, was stopped by this body. An operation was at length determined upon, with the father's consent. The patient's legs were tied as for lithotomy, and an incision made down upon the stone, which, when removed, was found to be a calculus, having a common bean for nucleus. The child had complained of pain and difficulty in micturition since the preceding Easter. It is probable that the child had passed the bean into its prepuce, whence it had slipped into the urethra and bladder.

In another case Dr. Leopold found a small calculus in the urethra, and was able to push it forward to the orifice of the urethra, which being enlarged by a slight incision, the calculus was readily removed.—*Casper's Wochenschrift*, 1850.

*** It seems scarcely possible that so large a substance as a bean could have slipped into the orifice of the urethra of a child, four years of age. It would almost lead one to suspect that a second party must have been herein concerned. The necessity which existed to enlarge the orifice of the urethra in the subsequent case tends to confirm this view. —*London Medical Gazette.*

